# Two Sulfur Glycoside Compounds Isolated from *Lepidium apetalum* Willd Protect NRK52e Cells against Hypertonic-Induced Adhesion and Inflammation by Suppressing the MAPK Signaling Pathway and RAAS

**DOI:** 10.3390/molecules22111956

**Published:** 2017-11-12

**Authors:** Peipei Yuan, Xiaoke Zheng, Meng Li, Yingying Ke, Yang Fu, Qi Zhang, Xiaolan Wang, Weisheng Feng

**Affiliations:** 1 College of Pharmacy, Henan University of Chinese Medicine, Zhengzhou 450046, China; 15136153630@163.com (P.Y.); limeng31716@163.com (M.L.); keyingying1988@sina.com (Y.K.); fuyang8692@163.com (Y.F.); zqfmaggily@163.com (Q.Z.); wxl_325@163.com (X.W.); fwsh@hactcm.edu.cn (W.F.); 2 Collaborative Innovation Center for Respiratory Disease Diagnosis and Treatment & Chinese Medicine Development of Henan Province, Zhengzhou 450046, China

**Keywords:** sulfur glycoside, hypertonic model, MAPK signaling pathway, RAAS, adhesion, inflammatory

## Abstract

*Lepidium apetalum* Willd has been used to reduce edema and promote urination. *Cis*-desulfoglucotropaeolin (*cis*-DG) and *trans*-desulfoglucotropaeolin (*trans*-DG) were isolated from *Lepidium apetalum* Willd, and caused a significant increase in cell viability in a hypertonic model in NRK52e cells. In the hypertonic model, *cis*-DG and *trans*-DG significantly promoted the cell viability of NRK52e cells and inhibited the elevation of Na^+^ in the supernatant, inhibited the renin-angiotensin-aldosterone (RAAS) system, significantly reduced the levels of angiotensin II (Ang II) and aldosterone (ALD), and lowered aquaporin-2 (AQP2) and Na^+^–K^+^ ATP content in renal medulla. After treatment with *cis*-DG and *trans*-DG, expression of calcineurin (CAN) and Ca/calmodulin-dependent protein kinase II (CaMK II) was decreased in renal tissue and Ca^2+^ influx was inhibited, thereby reducing the secretion of transforming growth factor-β (TGFβ), reversing the increase in adhesion and inflammatory factor E-selectin and monocyte chemotactic protein 1 (MCP-1) induced by high NaCl, while reducing oxidative stress status and decreasing the expression of cyclooxygenase-2 (COX2). Furthermore, inhibition of protein kinase C (PKC) expression also contributed to these improvements. The *cis*-DG and *trans*-DG reduced the expression of p-p44/42 MAPK, p-JNK and p-p38, inhibited the phosphorylation of the MAPK signaling pathway in NRN52e cells induced by high salt, decreased the overexpression of p-p38 and p-HSP27, and inhibited the overactivation of the p38-MAPK signaling pathway, suggesting that the p38-MAPK pathway may play a vital role in the hypertonic-induced adhesion and inflammatory response. From the results of this study, it can be concluded that the mechanism of *cis*-DG and *trans*-DG may mainly be through inhibiting the p38-MAPK signaling pathway, inhibiting the excessive activation of the RAAS system, and thereby reducing adhesion and inflammatory factors.

## 1. Introduction

*Lepidium apetalum* Willd was first described in the Eastern Han Chinese “Shen Nong Ben Cao Jing”. It is a commonly used Chinese medicine in the treatment of edema, cough and asthma. *Lepidium apetalum* Willd belongs to the Brassicaceae family. The dry mature seeds of this plant have been used in traditional Chinese medicine (TCM) to relieve cough, prevent asthma, reduce edema and promote urination. Several studies have reported that it contains various types of secondary metabolites, such as oils, flavonoids, sterols and cardiac glycosides [1,2,3], and shows antioxidant [1], antibacterial [2] and beneficial cardiac [4] activities. Clinical research suggests that it is useful for heart failure [5,6]. Therefore, studies of the phytochemical activity and structure–activity relationships of constituents of *Lepidium apetalum* Willd were developed in our lab.

According to the effect on reducing edema and promoting urination of *Lepidium apetalum* Willd, we tried to establish the corresponding cell model of hypertonic stress to investigate the mechanism at the molecular level. When the extracellular environment is hypertonic, cells lose water and consequently, shrink. To counteract this, cells increase their sodium uptake in order to lose less water. However, an increase in intracellular ionic concentration is harmful to the cell. Hypertonic stress leads to a decrease in cell viability, and intracellular and extracellular ionic strength may change. At the same time, due to the hypertonic effect, the intracellular Na^+^ level rises, renin-angiotensin-aldosterone system (RAAS) will be activated and the expression of cellular aquaporin-2 (AQP2) increases. The renin angiotensin aldosterone system (RAAS) plays an important role in blood pressure regulation, fluid volume and sodium balance, mainly including renin, angiotensin converting enzyme (ACE), angiotensin II (Ang II) and aldosterone (ALD). Excessive activation of the RAAS system can not only lead to vasoconstriction and sodium water retention, and cause high blood pressure, it contributes to the pathogenesis of a variety of clinical conditions, including progression of kidney disease, and leads to cardiac and vascular remodeling that affects cardiac function and reduces vascular elasticity [7,8]. Angiotensin II increases blood pressure by stimulating the Gq protein in vascular smooth muscle cells (which in turn activates an IP3-dependent mechanism leading to a rise in intracellular calcium levels and ultimately causing contraction). In addition, angiotensin II acts at the Na/H^+^ exchanger in the proximal tubules of the kidney to stimulate Na^+^ reabsorption and H^+^ excretion, which is coupled to bicarbonate reabsorption. In the adrenal cortex, angiotensin II acts to cause the release of aldosterone. Aldosterone acts on the tubules (e.g., the distal convoluted tubules and the cortical collecting ducts) in the kidneys, causing them to reabsorb more sodium and water from the urine. This ultimately results in an increase in blood volume and, therefore, increases blood pressure. As the main peptide hormone that causes vasoconstriction and a subsequent increase in blood pressure, angiotensin II also causes the occurrence of an inflammatory response [9]. Under normal conditions, pro-inflammatory cytokines and anti-inflammatory factors maintain a dynamic equilibrium with each other. After being subjected to external stimuli such as hypertonic stress, RAAS is activated, the level of angiotensin II increases, and the dynamic balance is broken, leading to a series of pathophysiological changes. This leads to an increase in the release of inflammatory factors, including transforming growth factor-β (TGFβ), monocyte chemotactic protein 1 (MCP-1), E-selectin and cyclooxygenase-2 (COX2). MAPKs are involved in directing cellular responses to a diverse array of stimuli, such as mitogens, osmotic stress, heat shock and pro-inflammatory cytokines. They regulate cell functions including proliferation, gene expression, differentiation, mitosis, cell survival and apoptosis. MAPKs are evolutionarily conserved signaling proteins present in all eukaryotes. They are activated by substantially diverse extracellular stimuli (e.g., osmotic stress), and the activation of multiple MAPK pathways orchestrates fundamental cellular processes (e.g., proliferation, growth, survival, migration, gene expression, cell cycle control and apoptosis) [10]. As the main effector cells of interstitial inflammatory injury, the surface membrane receptor CD40 of renal tubular epithelial cells binds to ligand CD154 in order to phosphorylate the MAPK signaling pathway to synthesize TGFβ, MCP-1 and other inflammatory factors, and upregulate COX2 expression [11,12]. These inflammatory factors are important regulatory factors in the inflammatory response of the kidneys. Many experiments have confirmed that MCP-1 has chemotactic activity in vivo, activates monocytes and macrophages, increases intracellular Ca^2+^ concentration, leads to the production and release of superoxide anions and upregulates the expression of adhesion factors such as E-selectin. While the increase in the concentration of Ca^2+^ in the body will directly lead to activation of protein kinase C (PKC), it will also activate the Ca^2+^/calmodulin-dependent protein kinases II -Calcineurin (CaMK II-CAN) signaling pathway. The CaMK II-CAN signaling pathway plays an important role in osmotic regulation, and the extracellular Ca^2+^ influx caused by osmotic response will further activate the CaMK II-CAN signaling pathway. Its downstream nuclear factor of activated T-cells 5, also known as NFAT5, is a human gene that encodes a transcription factor that regulates the expression of genes involved in osmotic stress [13,14]. Therefore, the activation of the CaMK II-CAN signaling pathway indicates the occurrence of hypertonic stress.

In the early stages of our group’s research, a large number of valuable monomer components were isolated from *Lepidium apetalum* Willd. The two sulfide glycoside compounds isolated from the 20% ethanol elution fraction were racemates and were isolated for the first time from *Lepidium apetalum* Willd [15]. After active screening, the two sulfide glycosides had a significant effect on the hypertonic model in NRK52e cells induced by high NaCl, suggesting that they may play a role in the treatment of kidney injury and other diseases (including nephritis, renal fibrosis and hypertension). Therefore, to further explore the mechanism of action of the two sulfide glycosides, we conducted this experiment to provide the basis of the discovery of new drugs with kidney damage-related diseases.

## 2. Results

### 2.1. *Cis*-DG and *Trans*-DG Increased the Cell Survival Rate in a Hypertonic Model in NRK52e Cells

The MTT assay was used to monitor cell viability in response to *cis*-desulfoglucotropaeolin (*cis*-DG) and *trans*-desulfoglucotropaeolin (*trans*-DG) treatment of the hypertonic model. As shown in Table 1, the cell survival rate of NRK52e cells was significantly decreased in the high-NaCl group compared with the normal control group (Control) (*p* < 0.01). While the *cis*-DG and *trans*-DG groups had a significantly increased cell survival rate compared with the high-NaCl group (*p* < 0.01), there was no difference between the *cis*-DG and *trans*-DG groups (*p* > 0.05), which indicated that *cis*-DG and *trans*-DG promoted the cell survival rate in the hypertonic model. 

### 2.2. *Cis*-DG and *Trans*-DG Decreased Ion Concentration in the Hypertonic Model in NRK52e Cells

When the extracellular environment is hypertonic, intracellular and extracellular ionic strength may change. An increase in intracellular ionic concentration is harmful to the cell. As shown in Table 2, the level of Na^+^ and Cl^−^ were significantly increased in the high-NaCl group (*p* < 0.01), and the level of K^+^ was decreased (*p* < 0.01). *Cis*-DG and *trans*-DG could reverse the level of Na^+^ (*p* < 0.01 and *p* < 0.05), but there was a more-significant decrease in the *cis*-DG group compared with the *trans*-DG group (*p* < 0.01). This result may be related to the difference of configuration, and the *cis*-configuration decreased more in the level of Na^+^.

### 2.3. *Cis*-DG and *Trans*-DG Inhibited RAAS Overactivation in the Hypertonic Model in NRK52e Cells

As a hormone system that regulates blood pressure and fluid balance, RAAS plays a vital role in the organism. In this research, we tested the components of the RAAS system, including renin, Ang II, ACE and ALD. From the results, we found that *cis*-DG and *trans*-DG could decrease the levels of Ang II and ALD (*p* < 0.01 or *p* < 0.05), as shown in Table 3, and reverse the situation of overactivation of RAAS, which suggested that the two compounds suppressed RAAS mainly through Ang II and ALD.

### 2.4. *Cis*-DG and *Trans*-DG Downregulated AQP2 and Na^+^–K^+^ ATP Expression in the Hypertonic Model in NRK52e Cells

AQP2 and Na^+^–K^+^ ATPs maintain the balance of water metabolism in the body. As shown in Table 4, in the high-NaCl group, cellular AQP2 and Na^+^–K^+^ ATP levels increased remarkably (*p* < 0.05), while *cis*-DG and *trans*-DG decreased cellular AQP2 and Na^+^–K^+^ ATP levels (*p* < 0.01 or *p* < 0.05). From the result, *cis*-DG decreased Na^+^–K^+^ ATP levels (*p* < 0.01) and *trans*-DG decreased Na^+^–K^+^ ATP levels (*p* < 0.05).

### 2.5. *Cis*-DG and *Trans*-DG Reduced CaMK II and CAN Expression in the Hypertonic Model in NRK52e Cells

As shown in Table 5, in the high-NaCl group, cellular CaMK II and CAN expression increased remarkably (*p* < 0.01), while *cis*-DG and *trans*-DG decreased cellular CaMK II and CAN significantly (*p* < 0.01).

### 2.6. *Cis*-DG and *Trans*-DG Reduced the Expression of Adhesion and Inflammatory Factors in the Hypertonic Model in NRK52e Cells

As shown in Table 6 and Table 7, high NaCl induced hypertonic stimulation, led to TGF-β and MCP-1 overexpression, suppressed adiponectin (APN) expression (*p* < 0.01), activated monocytes and macrophages, increased intracellular Ca^2+^ concentration and increased superoxide anion production and release, which resulted in PKC activation and an increase in E-selectin and COX2 expression (*p* < 0.01).

*Cis*-DG and *trans*-DG reversed TGF-β and MCP-1 overexpression and increased APN expression, but decreased PKC, E-selectin and COX2 expression (*p* < 0.01), thus alleviating the adhesion and inflammatory response induced by hypertonic stress.

### 2.7. *Cis*-DG and *Trans*-DG Inhibited the Overexpression of the MAPK and p38-MAPK Signaling Pathways in the Hypertonic Model in NRK52e Cells

**Figure 1:**
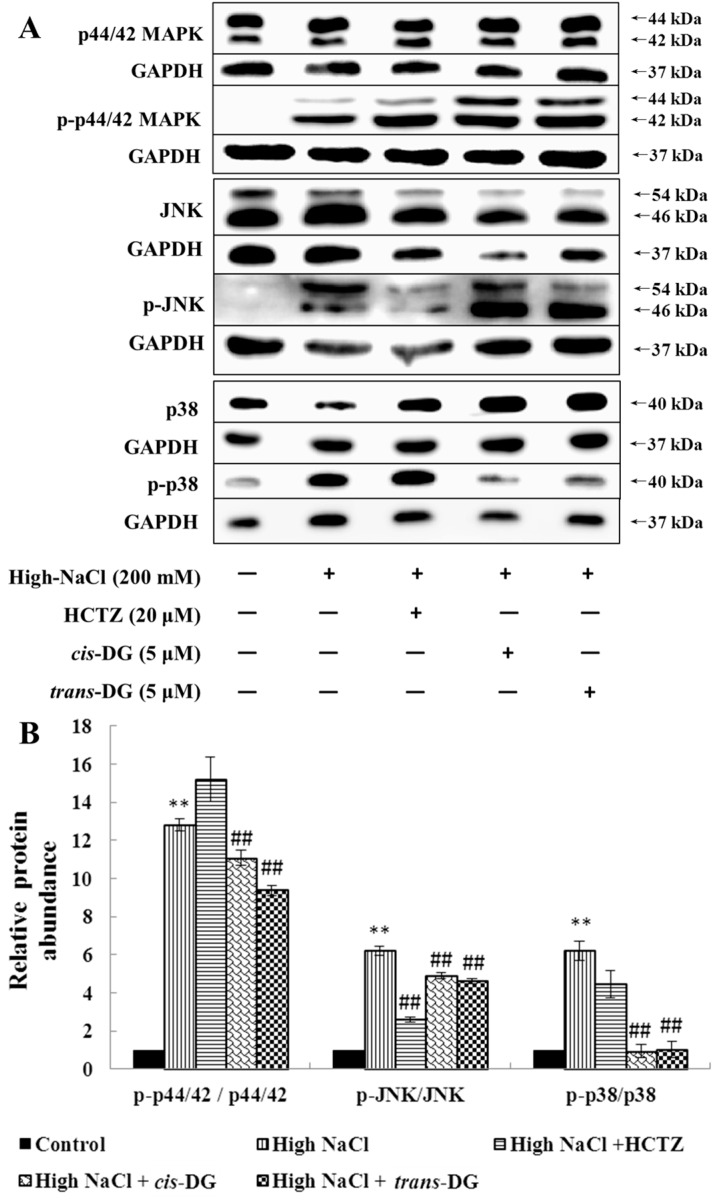
Influence of *cis*-DG and *trans*-DG (5 μM) on the MAPK signaling pathways in the hypertonic model in NRK52e cells for 6 h (*n* = 3). (**A**) The expression of p-p44/42 MAPK, p-JNK, p-p38, p44/42 MAPK, JNK and p38 measured by Western blot; (**B**) Relative protein abundance of MAPK signaling pathways proteins [(p-p44/42/GAPDH)/(p44/42/GAPDH), (p-JNK/GAPDH)/(JNK/GAPDH), (p-p38/GAPDH)/(p38/GAPDH)], analysed using SPSS 20.0 (IBM, New York, NY, USA), repeated three times. Note: significant difference versus control group: ** *p* < 0.01; significant difference versus high-NaCl group: ^##^ *p* < 0.01.

As shown in Figure 1, high NaCl-induced hypertonic stimulation resulted in p-p44/42 MAPK, p-c-Jun N-terminal kinase (p-JNK) and p-p38 overexpression (*p* < 0.01), indicating that the MAPK signaling pathway was mainly activated by the phosphorylated key proteins. *Cis*-DG and *trans*-DG suppressed the phosphorylation of the MAPK signaling pathway (*p* < 0.01), and showed the greatest improvement on p-p38.

**Figure 2:**
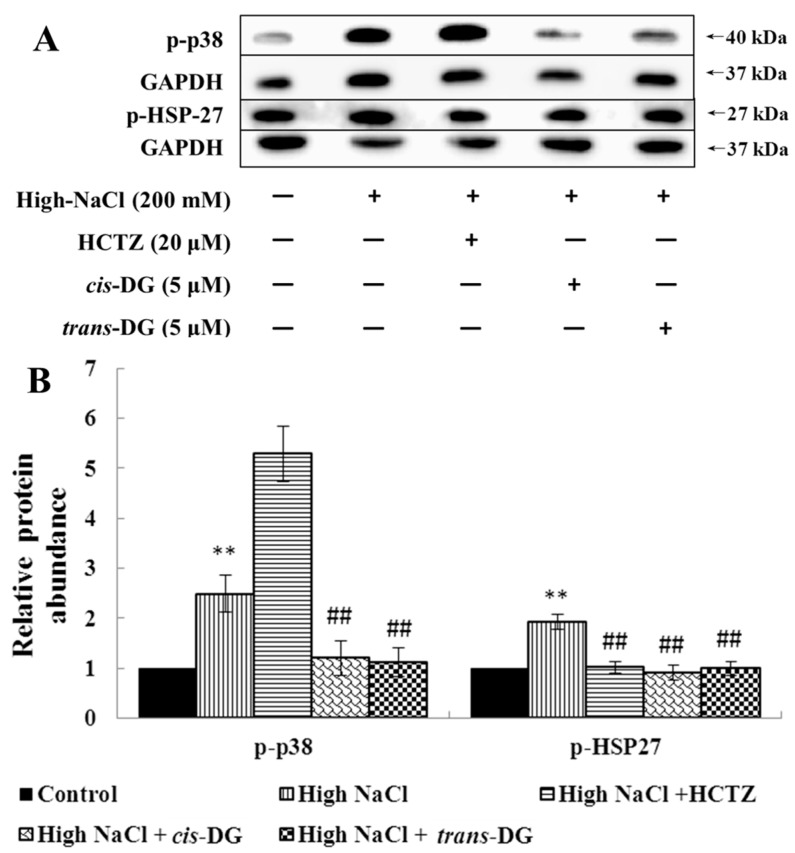
Influence of *cis*-DG and *trans*-DG (5 μM) on the p38-MAPK signaling pathways in the hypertonic model in NRK52e cells for 6 h (*n* = 3). **A**: The expression of p-p38 and p-HSP27 measured by Western blot; **B**: Relative protein abundance of p38-MAPK signaling pathway proteins (p-p38/GAPDH, p-HSP27/GAPDH), analysed using SPSS 20.0, repeated three times. Note: significant difference versus control group: ** *p* < 0.01; significant difference versus high-NaCl group: or ^##^ *p* < 0.01.

Meanwhile, as shown in Figure 2, p-p38 and p-heat shock protein 27 (p-HSP27) overexpression (*p* < 0.01) showed that the p38-MAPK signaling pathway was activated significantly. The intervention of *cis*-DG and *trans*-DG inhibited the overactivation of the p38-MAPK signaling pathway (*p* < 0.01), indicating that the p38-MAPK signaling pathway may play a key role in the hypertonic model.

## 3. Discussion

*Lepidium apetalum* Willd in the ancient Materia Medica was shown to have significant benefits of inducing diuresis for removing edema, reducing edema and promoting urination. Therefore, the effect of *cis*-DG and *trans*-DG was investigated for hypertonic stress in vitro, similar to the renal-injury or salt-sensitive-hypertension models.

The kidney undertakes three physiological functions in the body. Firstly, it is an important excretory organ for metabolic excretion in the body, maintaining the internal environment through the urine to produce metabolic end products, and excreting excess substances of the body to the outside [16]. Secondly, the kidney can regulate the water, electrolyte and acid–base equilibrium [17]. The regulation of moisture by the kidney depends on antidiuretic hormones. The regulation of Na^+^ and K^+^ ions depends on aldosterone. Thirdly, the kidney has an endocrine function and can secrete hormone-like substances, such as renin, bradykinin, prostaglandins, erythropoietin and others [18]. In short, the excretion of the kidneys, and regulation of electrolytes and the body-fluid balance, are accomplished through urine generation and excretion to maintain the normal osmotic pressure of the body. Epithelial cells isolated from mouse or rat medulla have become one of the best and most common model cells for cell osmotic regulation [19,20]. Therefore, in this study, we established the hypertonic model with high NaCl in NRK52e cells to mimic the renal injury of salt-sensitive hypertension in vivo. The RAAS system is an important neurohumoral mechanism that regulates water-sodium metabolism, and vasomotor and blood-pressure stability. Renin activates angiotensinogen into angiotensin I, and ACE can split angiotensin I into active angiotensin II, which can cause vasoconstriction and lead to high blood pressure. In addition, angiotensin II can also promote the synthesis and secretion of aldosterone, thus causing water-sodium retention, increasing blood circulation and increasing blood pressure [21,22]. Under the hypertonic stress, extracellular ionic strength rises, and cells lose water. The Na^+^ retention in vivo can lead to the expansion of blood volume, which leads to contraction of the arteries and an increase of peripheral resistance, eventually activating RAAS. The renin–angiotensin–aldosterone system (RAAS) is both a circulatory system and a local secretion system, and is one of the most important regulatory mechanisms of blood pressure. Its main effect is mediated by Ang II and its specific type 1 receptor (AT1R). When the signaling molecule Ang II activates its receptor AT1R on intraglomerular mesangial cells, this causes these cells to contract along with the blood vessels surrounding them and causes the release of aldosterone from the zona glomerulosa in the adrenal cortex [23]. Ang II not only plays an important role in vasoconstriction, but can also induce hypertension through oxidative stress and the inflammatory response [24]. Meanwhile, Ang II induces endothelial dysfunction by producing nitric oxide and mediating inflammation. Inflammation and endothelial dysfunction are the major risk factors of atherosclerosis and cardiovascular disease [25]. Excessive activation of the RAAS system is regarded as an important pathological link in the pathogenesis of primary hypertension, and the excessive secretion of plasma renin activity (PRA), Ang II and ALD in the system can raise blood pressure by promoting vasoconstriction and water-sodium reabsorption [26]. In this study, due to the increase in extracellular NaCl, the membrane receptor was activated and the expression of Na^+^–K^+^ ATPase increased, leading to overactivation of RAAS. *Cis*-DG and *trans*-DG can significantly inhibit the levels of Na^+^, Ang II and ALD in the RAAS system and alleviate the overactivation of RAAS (as shown in Table 2 and Table 3).

Osmotic stress arises from the difference between intracellular and extracellular osmolality. It induces cell swelling or shrinkage as a consequence of water influx or efflux, which threatens cellular activities. Mitogen-activated protein kinases (MAPKs) play central roles in signaling pathways in osmotic stress responses, including the regulation of intracellular levels of inorganic ions and organic osmolytes. MAPKs are evolutionarily conserved signaling proteins present in all eukaryotes. They are activated by substantially diverse extracellular stimuli (e.g., osmotic stress), and the activation of multiple MAPK pathways orchestrates fundamental cellular processes (e.g., proliferation, growth, survival, migration, gene expression, cell cycle control and apoptosis) [27,28,29,30]. The unique property of the MAPK signaling pathways is the three-layer phosphorylation cascade accomplished by different groups of kinases, which are referred to as “core signaling modules.” The most upstream group, the MAPK kinase kinases (MAP3Ks or MAPKKKs), phosphorylate Ser/Thr residues in the activation loop of MAPK kinases (MAP2Ks or MAPKKs). In turn, MAP2Ks phosphorylate MAPKs at the conserved Thr-X-Tyr motif in their activation loop. Extracellular signal-regulated kinase 1/2 (ERK1/2), p38-MAPK and c-Jun N-terminal kinase (JNK) are the vital members of MAPKs with involvement in osmotic stress signaling. The p38-MAPK signaling pathway plays an important role in the transduction of signal transduction molecules, which can be activated when phosphorylated. The activated p38-MAPK signaling pathway can continue to activate phosphorylation of downstream proteins, and for example, phosphorylate nuclear transcription factors to start gene transcription and to regulate the inflammatory response pathway [31]. In this study, hypertonic stimulation activated phosphorylation of the MAPK signaling pathway, and the p38-MAPK signaling pathway was also activated, leading to a series of inflammatory responses. *cis*-DG and *trans*-DG inhibited the activation of the MAPK signaling pathway, significantly reduced the phosphorylation levels of p-44/42 MAPK, p-JNK and p-p38, and decreased the expression of HSP27, indicating that *cis*-DG and *trans*-DG can inhibit excessive activity of the MAPK signaling pathway and improve the hypertonic stress state, mainly through the p38-MAPK signaling pathway (as shown in Figure 1 and Figure 2). Meanwhile, The Ca^2+^/CaMKII/CAN pathway regulates the activity of the transcription factors of the nuclear factor of activated T cells (NFAT) family. The NFAT family encompasses five individually encoded members. NFAT5, which differs in its structure from the other NFATs, is not regulated by calcium, but is activated in response to osmotic stress. NFATs are maintained in an inactive state in the cytosol of resting cells. Upon the stimulation of intracellular Ca^2+^ influx, CaM is activated by CaMKII and dephosphorylates the phosphorylation motifs from the N-terminus of NFATs, allowing NFATs to translocate to the nucleus where they collaborate with other transcription factors, such as AP-1, to encode a transcription factor that regulates the expression of genes involved in the osmotic stress [32]. This shows the important role of the CaMKII-CAN signaling pathway in osmotic stress regulation. Therefore, this study detected the expression of CaMKII and CAN. We found that *cis*-DG and *trans*-DG do have a significant regulatory role in the CaMKII-CAN signaling pathway (as shown in Table 5).

**Figure 3:**
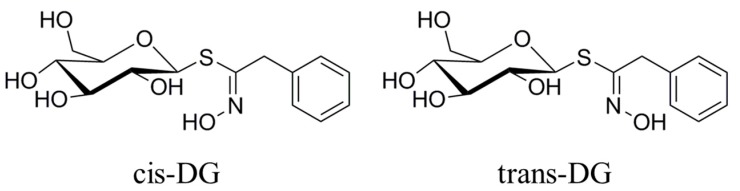
The structure of compounds *cis*-DG and *trans*-DG.

Kidney epithelial cells are the main effector cells of interstitial inflammatory injury. The combination of the surface membrane receptor, CD40, with the ligand, CD154, can activate the MAPK signaling pathway and synthesize IL-8 and MCP-1 inflammatory factors [14,33]. These inflammatory factors are important regulatory factors in the renal inflammatory response and renal interstitial fibrosis (RIF) process. The p38-MAPK pathway is one of the most important signaling pathways that can mediate cell survival under hypertonic stress conditions. Under high osmotic pressure, cells will activate some signaling pathways, including the p38-MAPK pathway, to induce osmotic stress-protection gene expression. Meanwhile, an increase in extracellular NaCl concentration can cause the production of reactive oxygen species (ROS) [34,35]. In general, ROS are destructive to cells, but in the hypertonic stress response, ROS can act as signaling molecules for osmotic protection. ROS participate in activation of the MAPK pathway and mediate increased expression of the COX2 protein in the hypertonic stress state [36]. At present, the biological function of TGF-β is mainly understood to be in inflammation, tissue repair and embryonic development. In recent years, researchers have found that TGF-β has an important regulatory role in cell growth, differentiation and immune function. MCP-1 is also known as a monocyte chemotactic and activating factor (MCAF), and belongs to the cysteine-cysteine subfamily (C-C subfamily) (β-subfamily). APN is an endogenous, biologically active polypeptide or protein secreted by adipocytes. It can reduce the adhesion of mononuclear cells in the early intervention of atherosclerotic lesions, and can also reduce the expression of various adhesion molecules (VCAM-1, ICAM-1, E-selectin) in endothelial cells. The results on TGF-β and MCP-1 as adhesion and inflammatory factors showed that increased extracellular NaCl concentration could cause increased secretion of TGF-β, promoting Ca^2+^ influx and intracellular inositol 1,3,4-trisphosphate (IP3) levels to increase and activate PKC. It also promoted the secretion of MCP-1, increased the chemotactic activity for activation of monocytes and macrophages, increased the cytoplasmic Ca^2+^ concentration, produced and released superoxide anions, and resulted in the uptake of adhesion molecules and E-selectin expression. Under a hypertonic stress state, intracellular APN expression decreased and activated monocytes and macrophages, resulting in a series of pathological changes. Hypertonic stimulation also increased COX2 expression and catalyzed prostaglandin (PG) synthesis in the inflammatory response. Intracellular elevated Ca^2+^ concentration leads to the activation of the CaMKII-CAN signaling pathway, further starting downstream regulation and transcription factor activity to undertake a series of regulatory responses. The experimental results showed that *cis*-DG and *trans*-DG can reduce the secretion of TGF-β and MCP-1 (as shown in Table 6) and decrease the concentration of Ca^2+^ in the cytoplasm, thus reducing the expression of PKC, COX2 and E-selectin, and alleviating the adhesion and inflammatory response (as shown in Table 7). It can be seen from Figure 3 that the forms of *cis*-DG and *trans*-DG are structurally similar, but the structures are not the same. Although the two compounds are similar in their mechanism of action, due to structural differences, there are differences in their roles, such as decreasing the expression of PKC and E-selectin and increasing the expression of APN. 

Several studies have reported that *Lepidium apetalum* Willd contains various types of secondary metabolites, such as oils, flavonoids, sterols and cardiac glycosides, but the basis of its efficacy are the flavonoids and flavonoid glycoside compounds [37]. A number of active ingredients were obtained from *Lepidium apetalum* Willd in our research group; among them, *cis*-DG and *trans*-DG significantly promoted the proliferation of NRK52e cells with a hypertonic model. As *Lepidium apetalum* Willd is used commonly as a treatment for edema, we presumed that *cis*-DG and *trans*-DG may also have a similar effect. In this study, the mechanism of *cis*-DG and *trans*-DG was studied from the cellular level, and it was found that *cis*-DG and *trans*-DG can inhibit the overactivation of RAAS and MAPK signaling pathways, thereby inhibiting the expression of adhesion factors and inflammatory factors and alleviating cell hypertonic stress, suggesting that *cis*-DG and *trans*-DG may have a potential therapeutic effect on edema. In the follow-up experiment, we will carry out the relevant animal experiments to explore its in-depth mechanism in vivo.

## 4. Materials and Methods 

### 4.1. Material

The seeds of *Lepidium apetalum* Willd were collected from Nanyang city in Henan Province, China, and were identified by Dr. Chen Suiqing and Dong Chengming (College of Pharmacy, Henan University of Chinese Medicine, Zhengzhou, China).

*Lepidium apetalum* Willd (8 kg) was prepared at 240 °C for 5.5 min and then extracted three times by water (80 L × 3) 1.5 h each at 100 °C. The solvent was evaporated under reduced pressure to get the crude extract. We adjusted the concentration of ethanol to 80% to make polysaccharide precipitate. The supernatant was concentrated, and then subjected to a Dianion HP-20 column followed by elution with water, 20% ethanol, 40% ethanol, 60% ethanol and 95% ethanol to give a water elution fraction (361 g), 20% ethanol elution fraction (71 g), 40% ethanol elution fraction (89 g), 60% ethanol elution fraction (67 g) and 95% ethanol elution fraction (28 g). The 20% ethanol eluting fractions (71 g) were applied to Toyopearl HW-40 column chromatography, and eluted with methanol–water (0:100 to 100:0) to give components A1 to A5. Component A2 (15 g) was chromatographed by ODS-18 reverse-phase column chromatography, and eluted with methanol–water (0:100 to 100:0) to give B1 to B7. Component B4 was subjected to Sephadex LH-20 column chromatography, and eluted with 70% methanol–water to give the components C1 to C3. C2 was applied to semi-preparative HPLC, and eluted with methanol–water (32:68) to get compound 1 (32 mg, tR = 40.6 min) and compound 2 (13 mg, tR = 86.6 min). Through the spectral data and physicochemical properties of the compounds, the two compounds were identified as *cis*-desulfoglucotropaeolin (*cis*-DG) and *trans*-desulfoglucotropaeolin (*trans*-DG), and the purity of these two compounds prepared in our laboratory was more than 98%, as determined by UV spectrophotometry [15] and as shown in Figure 3.

### 4.2. Cell Culture

Rat renal proximal tubular epithelial cell lines (NRK52e), which were purchased from ATCC (Rockville, MD, USA), were utilized in in-vitro experiments. The cells were cultured in Dulbecco’s Modified Eagle’s Medium (DMEM, HyClone, Logan, UT, USA) and supplemented with 10% Fetal Bovine Serum (FBS) (Gibco, Pittsburgh, PA, USA). A humidified incubator with 5% CO_2_ at 37 °C was used to culture the NRK52e cells.

### 4.3. Generation of High Salt-Induced Hypertonic Model in NRK52e Cells

The NRK52e cells were divided into the following groups: Control, the normal group without any treatment; High NaCl, the high-NaCl group, which consisted of 200 mmol/L NaCl; High NaCl + HCTZ pertained to the 20 μmol/L hydrochlorothiazide intervention group, which contained 200 mmol/L NaCl + 20 μmol/L HCTZ; High NaCl + *cis*-DG pertained to the 5 μmol/L *cis*-DG intervention group, which contained 200 mmol/L NaCl + 5 μmol/L *cis*-DG; High NaCl + *trans*-DG pertained to the 5 μmol/L *trans*-DG intervention group, which contained 200 mmol/L NaCl + 5 μmol/L *trans*-DG.

NRK52e cells were seeded in 96-well plates. The density of the cells in each plate was 2 × 10^4^ cells/mL. The cells were cultured at 37 °C in an atmosphere of 5% CO_2_. After the cells became adherent, we removed the original medium and washed the cells. The medium of the normal control group was replaced with DMEM containing 10% FBS. The medium of the model group was replaced with DMEM containing 10% FBS with 200 mmol/L NaCl. After the cells were cultured for 6 h, we detected the cell survival rate in each group to validate the model by methyl thiazolyl tetrazolium (MTT) assay.

### 4.4. The Experimental Groups

There were five groups in our study: the normal control group (Control), the high NaCl-induced model group (High NaCl) (treated with 200 mmol/L NaCl and cultivated for 6 h), the HCTZ group (High NaCl + HCTZ) (treated with 10 μmol/L HCTZ), the *cis*-DG group (treated with 5 μmol/L *cis*-DG) and the *trans*-DG group (treated with 5 μmol/L *trans*-DG).

NRK52e cells were seeded in 96-well plates. The density of the cells in each plate was 2 × 10^4^ cells/mL. The cells were cultured at 37 °C in an atmosphere of 5% CO_2_. After the cells became adherent, we removed the original medium and washed the cells. The medium of the normal control group was replaced with DMEM containing 10% FBS. The medium of the model group was replaced with DMEM containing 10% FBS with 200 mmol/L NaCl. The medium of the HCTZ group was replaced with DMEM containing 10% FBS with 200 mmol/L NaCl and 20 μmol/L HCTZ. The medium of the *cis*-DG group was replaced with DMEM containing 10% FBS with 200mmol/L NaCl and 5 μmol/L *cis*-DG. The medium of the *trans*-DG group was replaced with DMEM containing 10% FBS with 200 mmol/L NaCl and 5 μmol/L *trans*-DG. The cells were cultured for 6 h, and then carried on in the follow-up experiments.

### 4.5. Cell Viability Assay

To measure cell proliferation, a methyl thiazolyl tetrazolium (MTT) assay was used. After passing the incubation time of treatment of NRK52e cells with *cis*-DG and *trans*-DG, the medium was substituted with (200 µL) fresh media containing 20 µL of MTT solution (2 mg/mL in Phosphate Buffer Saline (PBS), and the cells were treated for an additional 4 h at 37 °C. Then, the media/MTT mixture was removed and 150 μL of DMSO was added to each well. After 10 min of shaking in the plate to dissolve the crystals, the absorbance of each well was measured using a microplate reader at 490 nm. MTT solution with DMSO (without the cells and medium) was used as a blank control [38]. Finally, cell survival was calculated as: survival (%) = (mean experimental absorbance/mean control absorbance) × 100% [39].

### 4.6. Enzyme-Linked Immunosorbent Assay

For the ELISA assay, a 100 mm × 20 mm Petri dish was used. The density of cells in each dish was 2 × 10^5^ cells/mL. After treatment with *cis*-DG and *trans*-DG for 6 h, the culture medium was collected from each petri dish and centrifuged. The supernatants were collected and used to detect the levels of renin (E-EL-R0030c, Elabscience Biotechnology Co. Ltd., Wuhan, China), ACE (E-EL-R2401c, Elabscience Biotechnology Co. Ltd.), Ang II (E-EL-R1430c, Elabscience Biotechnology Co. Ltd.), ALD (E-EL-0070c, Elabscience Biotechnology Co. Ltd.), MCP-1 (E-EL-R0633c, Elabscience Biotechnology Co. Ltd.) and TGF-β (E-EL-R0084c, Elabscience Biotechnology Co. Ltd.) according to the respective manufacturer’s instructions. The cells in each group were also collected to extract total protein, cytoplasmic protein and nuclear protein according to the manufacturer’s instructions (P0028, Beyotime Institute of Biotechnology, Shanghai, China). The protein solution was used in Western blot experiments after its concentration was determined using the BCA protein assay kit (Solarbio, No.20170815, Beijing Solarbio Science & Technology Co. Ltd., Beijing, China). After measuring its concentration, we used the extract to determine the activities of AQP2 (CSB-E08243r), Na^+^–K^+^ ATPs (A070-2, Nanjing Jiancheng Bioengineering Institute, Nanjing, China), CAN (E-EL-R0134c, Elabscience Biotechnology Co. Ltd.), CaMKII (CSB-E10034r, Cusabio Biotech Co. Ltd., Wuhan, China), APN (AK0017APR19018, Elabscience Biotechnology Co. Ltd.), PKC (E-EL-R0815c, Elabscience Biotechnology Co. Ltd.), COX2 (E-EL-R0792c, Elabscience Biotechnology Co. Ltd.) and E-selectin (E-EL-R0893c, Elabscience Biotechnology Co. Ltd.). Summary of the experiment is as follows: (1) add 100 μL standard or sample to each well. Incubate 90 min at 37 °C; (2) remove the liquid. Add 100 μL biotinylated detection antibody (Ab). Incubate 1 h at 37 °C; (3) aspirate and wash 3 times; (4) add 100 μL horseradish peroxidase (HRP) conjugate. Incubate 30 min at 37 °C; (5) aspirate and wash 5 times; (6) add 90 μL substrate reagent. Incubate 15 min at 37 °C; (7) add 50 μL stop solution. Read at 450 nm immediately; (8) according to the standard curve line and the OD values of the samples, calculate the results.

### 4.7. Western Blot Analysis

Equal amounts (20 μg) of nuclear and cytoplasmic extracts were used for 12% SDS–PAGE analysis, which were then transferred to a polyvinylidene difluoride membrane and then blocked in 5% nonfat milk in Tris-buffered saline with Tween-20 (TBST, 0.1%) for 2 h at room temperature and then immunoblotted with the corresponding primary antibodies and incubated overnight at 4 °C. After washing with TBST (5 × 5 min), the membranes were incubated with the appropriate secondary antibodies for 1 h at room temperature and then detected using a ChemiDoc XRS system (Bio-Rad, Hercules, CA, CA, USA). Finally, Image J (NIH, Rockville, MD, USA) was used to quantify the protein bands.

### 4.8. Statistical Analyses

All analyses were performed using SPSS 20.0 (IBM, New York, NY, USA). The quantitative data were expressed as the mean ± SEM ($\bar{x}$ ± s, *n* = 5). Statistical significance was assessed in comparison with the respective control for each experiment using one-way ANOVA. *p* < 0.05 was accepted as significant.

## 5. Conclusions

The mechanism by which treatment with *cis*-DG and *trans*-DG reverses osmotic stress in NRK52e cells may involve inhibition of adhesion and the inflammatory response by suppression of the MAPK signaling pathways (especially the p38-MAPK signaling pathway) and RAAS.

## Figures and Tables

**Table 1:** Effect of *cis*-desulfoglucotropaeolin (*cis*-DG) and *trans*-desulfoglucotropaeolin (*trans*-DG) (5 μM) on the cell survival rate in the hypertonic model in NRK52e cells for 6 h ($\bar{x}$ ± s, *n* = 5).

Groups	Cell Survival Rate (%)
Control	100.00 ± 6.16
High NaCl	78.14 ± 6.08 **
High NaCl + HCTZ ^1^	89.35 ± 3.72 ^#^
High NaCl + *cis*-DG	96.08 ± 5.59 ^##^
High NaCl + *trans*-DG	90.93 ± 7.18 ^##^

Note: significant difference versus control group: ** *p* < 0.01; significant difference versus high-NaCl group: ^#^ *p* < 0.05 or ^##^ *p* < 0.01. ^1^ HCTZ: Hydrochlorothiazide.

**Table 2:** Influence of *cis*-DG and *trans*-DG (5 μM) on ions in the hypertonic model in NRK52e cells for 6 h ($\bar{x}$ ± s, *n* = 5).

Groups	Na^+^ (mmol/L)	K^+^ (mmol/L)	Cl^−^ (mmol/L)
Control	159.18 ± 3.39	5.28 ± 0.03	119.89 ± 1.57
High NaCl	248.95 ± 7.07 **	4.86 ± 0.04 **	194.76 ± 1.71 **
High NaCl + HCTZ	175.25 ± 16.02 ^##^	5.09 ± 0.09 ^##^	190.51 ± 0.86 ^##^
High NaCl + *cis*-DG	198.42 ± 4.15 ^##^	4.97 ± 0.09	193.11 ± 3.33
High NaCl + *trans*-DG	225.44 ± 19.39 ^#^	4.93 ± 0.04	193.02 ± 1.57

Note: significant difference versus control group: ** *p* < 0.01; significant difference versus high-NaCl group: ^#^ *p* < 0.05 or ^##^ *p* < 0.01.

**Table 3:** Influence of *cis*-DG and *trans*-DG (5μM) on RAAS in the hypertonic model in NRK52e cells for 6 h ($\bar{x}$ ± s, *n* = 5).

Groups	Renin (pg/mL)	Angiotensin II (Ang II) (pg/mL)	Angiotensin Converting enzyme (ACE) (U/L)	Aldosterone (ALD) (pg/mL)
Control	18.62 ± 0.51	119.2 ± 9.58	34.91 ± 0.25	95.02 ± 14.15
High NaCl	13.89 ± 0.57 **	158.63 ± 8.47 **	34.41 ± 2.06	124.84 ± 13.54 **
High NaCl + HCTZ	14.97 ± 2.57	130.64 ± 16.86 ^#^	31.13 ± 4.34	92.42 ± 15.77 ^#^
High NaCl + *cis*-DG	13.12 ± 1.81	96.58 ± 11.01 ^##^	25.75 ± 1.66	93.72 ± 17.21 ^#^
High NaCl + *trans*-DG	12.74 ± 2.33	109.11 ± 18.88 ^##^	27.62 ± 2.44	92.31 ± 8.86 ^#^

Note: significant difference versus control group: ** *p* < 0.01; significant difference versus high-NaCl group: ^#^ *p* < 0.05 or ^##^ *p* < 0.01.

**Table 4:** Influence of *cis*-DG and *trans*-DG (5 μM) on ions in the hypertonic model in NRK52e cells for 6 h ($\bar{x}$ ± s, *n* = 5).

Groups	Aquaporin-2 (AQP2) (pg/mL)	Na^+^–K^+^ ATPs (μmol/mL)
Control	129.15 ± 2.43	5.91 ± 0.35
High NaCl	159.16 ± 5.85 *	7.22 ± 0.50 *
High NaCl + HCTZ	124.96 ± 17.04 ^##^	6.15 ± 0.70 ^#^
High NaCl + *cis*-DG	115.12 ± 2.43 ^##^	5.39 ± 0.53 ^##^
High NaCl + *trans*-DG	112.39 ± 2.34 ^##^	6.21 ± 0.81 ^#^

Note: significant difference versus control group: * *p* < 0.05; significant difference versus high-NaCl group: ^#^ *p* < 0.05 or ^##^ *p* < 0.01.

**Table 5:** Influence of *cis*-DG and *trans*-DG (5 μM) on Ca/calmodulin-dependent protein kinase II (CaMK II) and calcineurin (CAN) in the hypertonic model in NRK52e cells for 6 h ($\bar{x}$ ± s, *n* = 5).

Groups	CaMK II (pg/mL)	CAN (ng/mL)
Control	369.46 ± 92.07	5.14 ± 0.45
High NaCl	981.84 ± 122.98 **	10.82 ± 0.08 **
High NaCl + HCTZ	648.90 ± 85.28 ^##^	4.99 ± 0.11 ^##^
High NaCl + *cis*-DG	340.84 ± 97.72 ^##^	6.26 ± 1.34 ^##^
High NaCl + *trans*-DG	461.22 ± 25.74 ^##^	6.32 ± 1.75 ^##^

Note: significant difference versus control group: ** *p* < 0.01; significant difference versus high-NaCl group: ^##^ *p* < 0.01.

**Table 6:** Influence of *cis*-DG and *trans*-DG (5 μM) on transforming growth factor-β (TGFβ), monocyte chemotactic protein 1 (MCP-1), E-selectin and adiponectin (APN) in the hypertonic model in NRK52e cells for 6 h ($\bar{x}$ ± s, *n* = 5).

Groups	TGFβ (pg/mL)	MCP-1 (ng/mL)	APN (pg/mL)
Control	10.43 ± 3.28	0.04 ± 0.02	24.01 ± 3.41
High NaCl	71.61 ± 14.34 **	0.27 ± 0.01 **	7.12 ± 3.46 **
High NaCl + HCTZ	23.4 ± 2.48 ^##^	0.18 ± 0.001 ^##^	20.22 ± 5.08 ^##^
High NaCl + *cis*-DG	28.03 ± 5.07 ^##^	0.1 ± 0.001 ^##^	7.40 ± 3.29
High NaCl + *trans*-DG	10.89 ± 3.77 ^##^	0.07 ± 0.01 ^##^	22.6 ± 0.76 ^##^

Note: significant difference versus control group: ** *p* < 0.01; significant difference versus high-NaCl group: ^##^ *p* < 0.01.

**Table 7:** Influence of *cis*-DG and *trans*-DG (5 μM) on protein kinase C (PKC), E-selectin and cyclooxygenase-2 (COX2) in the hypertonic model in NRK52e cells for 6 h ($\bar{x}$ ± s, *n* = 5).

Groups	PKC (pg/mL)	E-Selectin (pg/mL)	COX2 (pg/mL)
Control	17.45 ± 2.23	12.45 ± 1.93	32.19 ± 9.13
High NaCl	68.64 ± 1.77 **	35.91 ± 4.83 **	48.69 ± 2.65 **
High NaCl + HCTZ	52.23 ± 11.94 ^#^	40.94 ± 5.29 **	27.24 ± 7.36 ^##^
High NaCl + *cis*-DG	76.88 ± 6.63	24.43 ± 1.93 ^##^	27.45 ± 1.71 ^##^
High NaCl + *trans*-DG	17.90 ± 2.18 ^##^	15.42 ± 1.37 ^##^	26.98 ± 6.42 ^##^

Note: significant difference versus control group: ** *p* < 0.01; significant difference versus high-NaCl group: ^#^ *p* < 0.05 or ^##^ *p* < 0.01.

## Abbreviations

The following abbreviations are used in this manuscript:

Ab	antibody
ACE	Angiotensin converting enzyme
APN	Adiponectin
ALD	Aldosterone
Ang II	Angiotensin II
AQP2	Aquaporin-2
*cis*-DG	*Cis*-desulfoglucotropaeolin
CaMKII	Ca^2+^/calmodulin-dependent protein kinases II
CAN	Calcineurin
C-C subfamily	Cysteine-cysteine subfamily
COX2	Cyclooxygenase-2
ELISA	Enzyme-linked immunosorbent assay
FBS	Fetal Bovine Serum
HCTZ	Hydrochlorothiazide
HRP	horseradish peroxidase
HSP27	heat shock protein 27
IP3	Inositol 1,3,4-trisphosphate
JNK	c-Jun N-terminal kinase
MCP-1	Monocyte chemotactic protein 1
MTT	Methyl thiazolyl tetrazolium
PBS	Phosphate Buffer Saline
PG	Prostaglandin
PKC	Protein kinase C
PRA	Plasma renin activity
RAAS	renin-angiotensin-aldosterone system
RIF	Renal interstitial fibrosis
*trans*-DG	*Trans*-desulfoglucotropaeolin
TGFβ	Transforming growth factor-β
